# Supplementary material for: Stem cell-associated heterogeneity in Glioblastoma results from intrinsic tumor plasticity shaped by the microenvironment
Source: Nat Commun. 2019 Apr 16;10:1787. doi: 10.1038/s41467-019-09853-z (PMC6467886; doi:10.1038/s41467-019-09853-z)
Supplement: Supplementary file 1 — Supplementary Information [file 41467_2019_9853_MOESM1_ESM.pdf]

SUPPLEMENTARY INFORMATION

Stem cell-associated heterogeneity in Glioblastoma results from intrinsic tumor plasticity shaped by the microenvironment

Dirkse, Golebiewska et al.

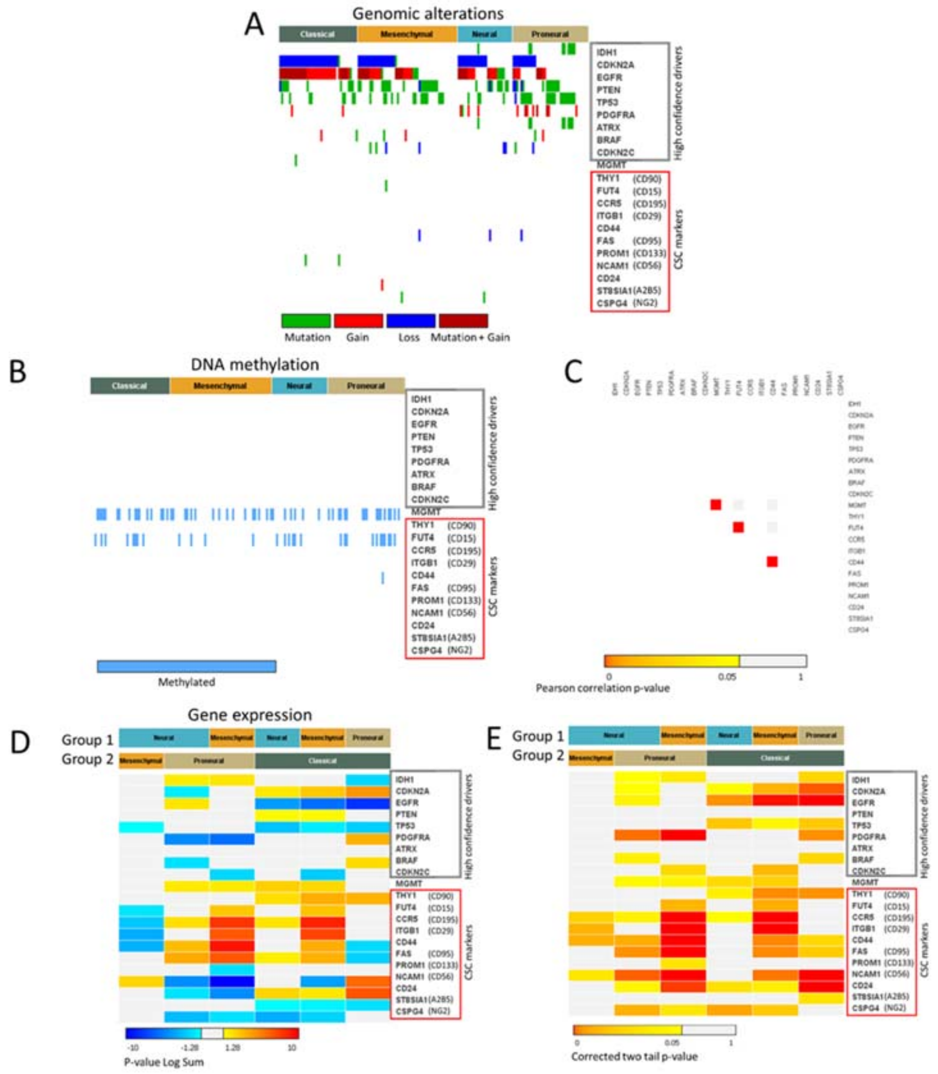

Supplementary Figure 1. Genomic alterations, DNA methylation and gene expression in GBM patients. A. Genetic aberrations and mutations in GBM patient samples. Analysis of genetic alterations in main cancer and selected stem cell-associate cell membrane markers in GBM. Heatmap showing an integrated value dimension for genomic alterations (mutation and/or copy number alteration) in the different GBM expression subgroups (Proneural, Neural, Mesenchymal and Classical) B. Heatmap showing promoter methylation of some genes in clinical samples. C. Heatmap of Pearson correlation significance calculated from promoter methylation data. Only the *FUT4* promoter displayed altered DNA methylation, which was not correlated to the *MGMT* promoter methylation (p_{value} = 0.086). D. Group comparison for CSC-associated gene expression in GBM molecular subgroups. GBM subgroups are shown in column headers. P_{value} log summary is depicted in left panel as follows: yellow-to-red is represented if values in Group 1 are significantly shifted. E. Statistical analysis of group comparisons (Corrected two tail p_{value}). Several markers were correlated with previously defined transcriptional subgroups *e.g.* CCR5 (CD195), ITGB1 (CD29), CD44 and FAS (CD95) are more expressed in the mesenchymal subtype, whereas PROM1 (CD133), NCAM (CD56) and CD24 are enriched in the proneural subtype.

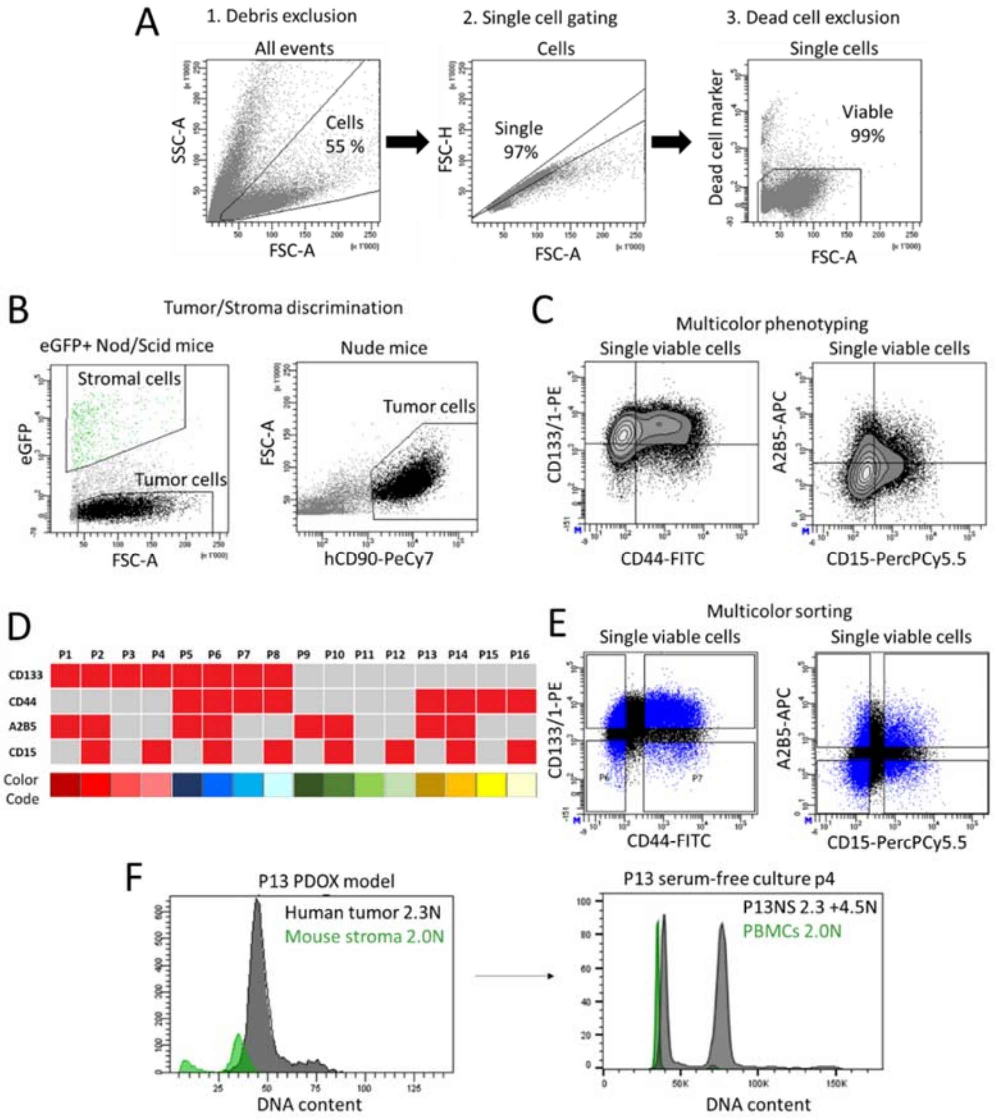

Supplementary Figure 2. Gating strategy for multicolor phenotypic analysis and sorting. A. The step-by-step gating strategy for FACS analysis is shown for the intracranial xenograft. The same strategy was used for all subsequent experiments using single viable cells. (1) Cells were distinguished from debris on the flow cytometric profile based on the Forward Scatter (FSC) and Side Scatter (SSC). (2) Cell doublets and aggregates were gated out based on their properties displayed on the FSC area (FSC-A) versus height (FSC-H) dot plot. (3) Dead cells were recognized by their strong positivity for the dead cell discrimination marker. B. In xenografts, human tumor cells were selected as the eGFP negative population (eGFP+ Nod/SCID mice) or hCD90 positive cells (nude mice and on-eGFP Nod/SCID mice). C. Multicolor phenotyping was performed by simultaneous staining for four CSC-associated markers: CD133, CD44, CD15, and A2B5. D. 16 subpopulations were distinguished based on the presence/absence of the expression of the four cell membrane markers. E. For multicolor sorting more stringent gates were applied to ensure no overlap between the 16 subpopulations. FACS-sorting gates were applied near the brightest and dimmest ends of the spectrum. F. Example of ploidy analysis of tumor cells in vivo in PDOX P13 model (left) and PDOX-derived short term serum-free cultures (P13NS passage 4). Mouse stroma and PBMCs were used as diploid control (2N) *in vivo* and *in vitro* respectively.

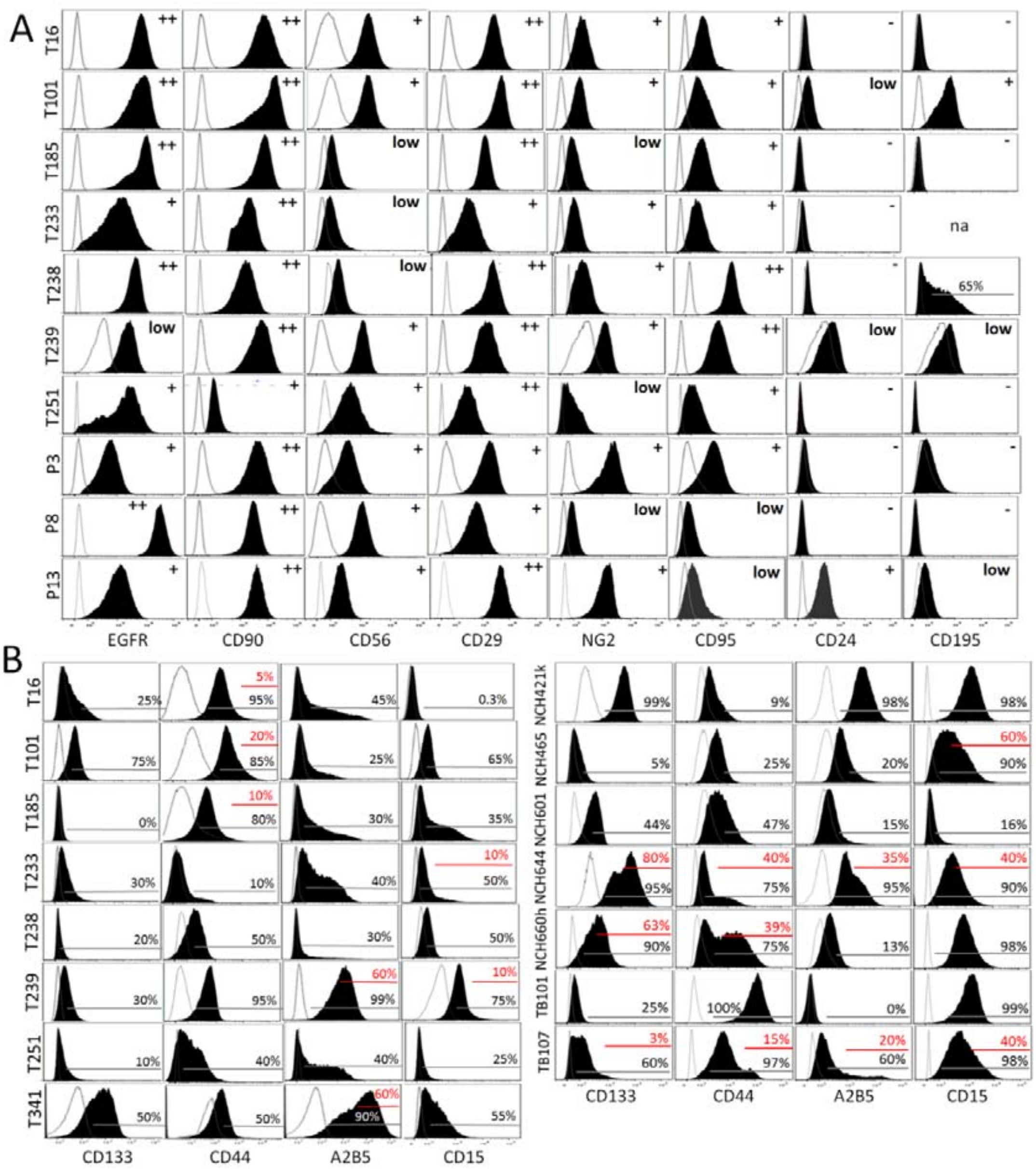

Supplementary Figure 3. CSC-associated marker expression profiles in GBM PDOXs and cultures. A. Flow cytometric analysis of tumor cells showing inter-patient heterogeneity for a panel of CSC-associated epitopes in GBM PDOXs. Expression was considered negative (-) when positive staining was equal to negative control, low when mean fluorescence intensity (MFI) of positive staining was less than 10x negative control (low), positive with 10-100x difference in MFI (+) and high when MFI was >100x higher compared to negative control (++). B. Flow cytometric analysis of tumor cells showing inter-patient heterogeneity for the CD133, CD44, CD15 and A2B5 epitopes in GBM PDOXs (left panels) and GBM cultures (right panels). Percentage of positive cells is indicated for all markers vs. negative control (black gating, positive vs. negative cells). For intratumoral heterogeneity discriminating negative, low and high expressing cells additional gating was applied (red gating). For defining 16 distinct subpopulations heterogeneity based on low vs. high expression levels (red gating) had priority over negative vs. positive discrimination (black gating) if applicable. See Supplementary Figure 2 for gating strategy. The PDOX models are defined based on histopathological features as angiogenic (P13), intermediate (P3, T16, T238, T341) or invasive phenotype (P8, T101, T185, T233, T239, T251)[1].

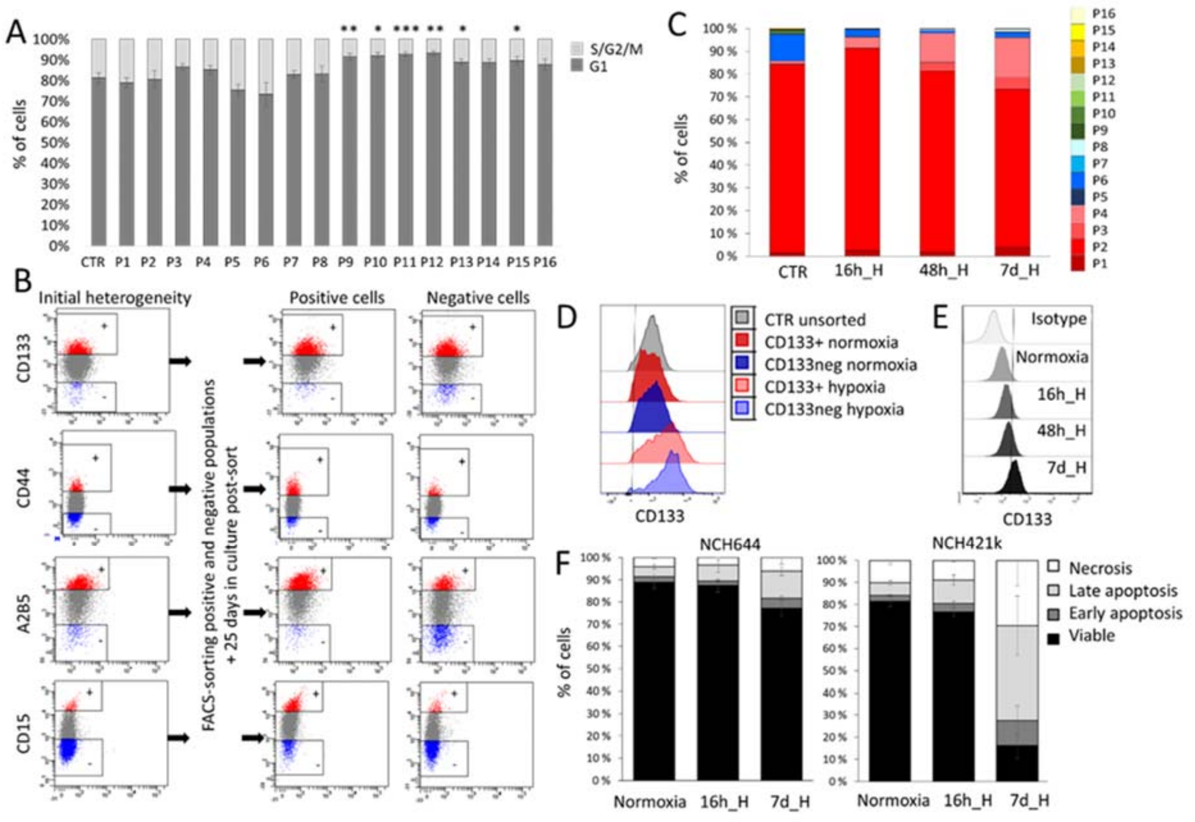

Supplementary Figure 4. Characterization of intratumoral CSC-associated heterogeneity. A. Cell cycle analysis of 16 subpopulations. Cells in S/G2/M cell cycle phase were distinguished from the G1 phase based on the Hoechst flow cytometric profile combined with multicolor phenotyping in viable cells (n = 3, mean +/- SEM, $*p_{value} \leq 0.05$; $**p_{value} \leq 0.01$; $***p_{value} \leq 0.001$, Student's t-test). B. Multipotency of NCH421k cells. NCH421k cells were stained with one epitope at a time (CD133, CD44, A2B5 and CD15). 500 positive and negative cells were FACS-sorted, cultured (n = 4) and re-phenotyped after 25 days for the presence of the same markers. All populations were able to recreate initial heterogeneity. Representative graphs are shown. C. Distribution of NCH421k subpopulations upon hypoxia. Cells were cultured in normoxia (N) and hypoxia (H) for 16h, 48h, and 7 days. Distribution of subpopulations is presented as a mean percentage of each subpopulation in viable single cells ($n_B = 3$; $n_T = 3$). See Supplementary Data 1C for statistics. D. CD133 positive and negative NCH421k cells were FACS-sorted, cultured in normoxia or hypoxia, and re-phenotyped for CD133 expression after 77 days. CD133 positive and negative population was able to reach similar normoxic and hypoxic-specific equilibrium in time. Due to slow growth of NCH421k cells in severe hypoxia earlier time point was not available. Black line discriminates between CD133 negative and positive cells. E. Expression of CD133 was analyzed in U87 adherent cultures in normoxia or hypoxia for 16h (16h_H), 48h (48h_H) and 7 days (7d_H). Black line discriminates between CD133 negative and positive cells. F. Apoptosis test of NCH644 and NCH421k cultures in normoxia or after 16h (16h_H) and 7 days (7d_H) hypoxia. Single cells were divided into four categories: viable (AnnexinV$^-$ PI$^-$), early apoptotic (AnnexinV$^+$ PI$^-$), late apoptotic (AnnexinV$^+$ PI$^+$) and necrotic (AnnexinV$^-$ PI$^+$). Bar plots represent % of cells (mean ± SEM, n_B=2-3, n_T=2-3, Student's t-test with Bonferroni multiple-significance-test correction; $p_{value}* < 0.05$, $p_{value}*** < 0.001$).

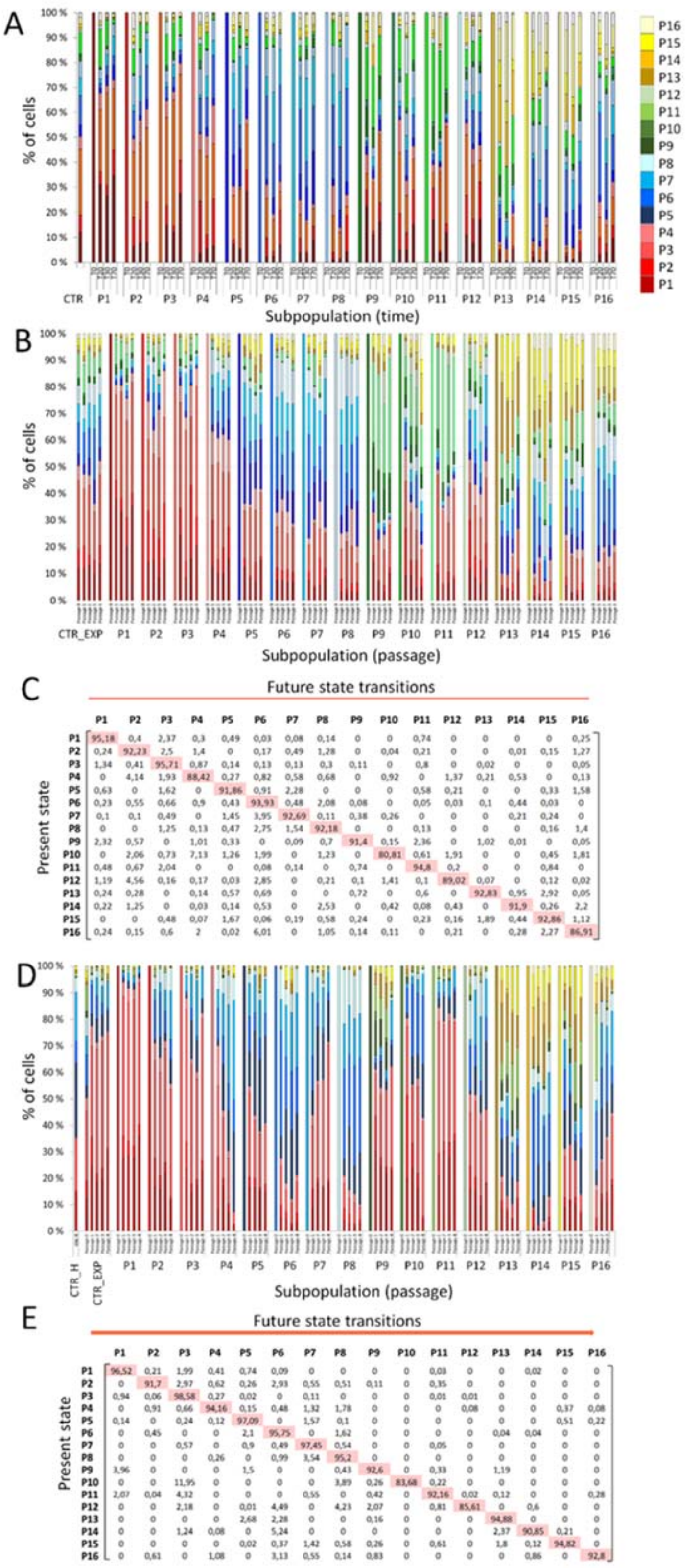

Supplementary Figure 5. GBM state transitions in normoxia and hypoxia. A. Distribution of 16 FACS-sorted subpopulations over time. Re-phenotyping was performed 20 (D20), 30 (D30) and 70 (D70) days after FACS (D0) for each subpopulation (n_B = 4; n_T = 3). The distribution of subpopulations is presented as a mean percentage of each subpopulation in viable single cells. Distribution of 16

subpopulations is presented for control cultures (CTR). See Supplementary Data 2A for statistics. B. Distribution of 16 subpopulations after single cell sorting (self-renewal test, passage 1-4). Re-phenotyping was performed at each self-renewal passage for each subpopulation (all single spheres gathered together in one pool, n=4). Distribution of subpopulations is presented as a mean percentage of each subpopulation in viable single cells. Viable single cells were sorted as a control (CTR_EXP). See Supplementary Data3A for statistics. C. Markov modelling of state transitions in normoxia. Probabilities of state transitions within one time-step (1 day) are presented for each subpopulation. Y axis displays subpopulations in the present state. X axis displays the probabilities of each subpopulation to change to respective phenotypes. D. Graphs represent distribution of 16 subpopulations after single cell sorting (self-renewal test, passage 1-4) cultivated in hypoxia. Re-phenotyping was performed at each self-renewal passage for each subpopulation (n = 4). Distribution of subpopulations is presented as a mean percentage of each subpopulation in viable single cells. Viable single cells were sorted as a control (CTR_EXP). See Supplementary Data 3B for statistics. Distribution of 16 subpopulations of control cultures after 60 days in hypoxia is presented as an environmental control (CTR_H). E. Markov modelling of state transitions in hypoxia. Probabilities of state transitions within one time-step (1 day) are presented for each subpopulation. Y axis displays subpopulations in the present state. X axis displays probabilities of each subpopulation to change to respective phenotypes.

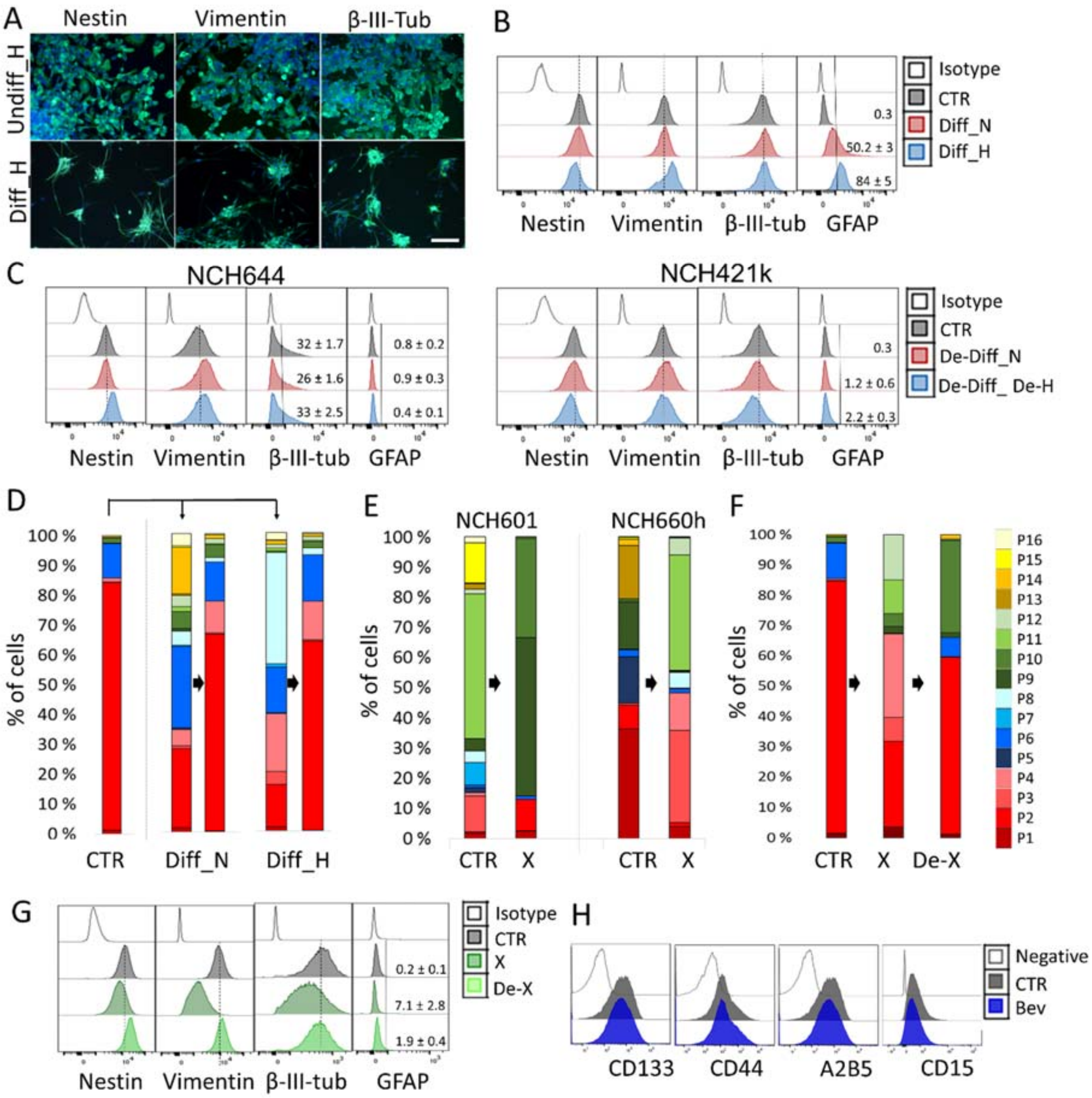

Supplementary Figure 6. Reversible adaptation upon differentiation and in vivo microenvironment. A. Examples of ICC after 14d of adherent cultures in stem cell (Undiff_H) or differentiation (Diff_H) conditions in hypoxia. (scale bar = 200 µm) B. Flow cytometric analysis of internal stem cell (Nestin, Vimentin) and differentiation markers (β-III-tubulin, GFAP) in control 3D NCH421k cultures (CTR), and differentiation (Diff) conditions after 14 days. N = normoxia, H = hypoxia (n = 3). Negative control for antibody staining is shown for each type of cells (Isotype). Black lines discriminate between negative and positive cells. Dotted line indicate mode expression in control cells. C. Flow cytometric analysis of internal stem cell (Nestin, Vimentin) and differentiation markers (β-III-tubulin, GFAP) in control 3D NCH644 and NCH421k cultures (CTR), and 14 day 3D cultures that were previously subjected to differentiation (De-Diff) conditions. N= normoxia, H= hypoxia (n_B = 3; n_T = 3). D. Distribution of NCH421k subpopulations upon differentiation (Diff) conditions, N = normoxia, H = hypoxia (n = 3). For each condition equilibrium is presented after 14 days of environmental change (left) and 14 days after consecutive change to original 3D stem cell conditions (right). NCH421k cultured as 3D spheres in normoxia are shown as control (CTR). See Supplementary Data 1E for

statistics. E. Graphs represent distribution of 16 subpopulations in xenografted NCH601 and NCH660h tumor cells (X). Normoxic cultures are shown as a control (CTR) (n = 3). F. Distribution of 16 subpopulations in xenografted NCH421k tumor cells (X) and 56 days after regrowth *in vitro* as 3D cultures (De-X) ($n_B = 2$; $n_T = 3$). NCH421k cultured in normoxia are shown as a control (CTR). See Supplementary Data 1G for statistics. G. Flow cytometric analysis of internal stem cell (Nestin, Vimentin) and differentiation markers (β-III-tubulin, GFAP) in control NCH421k cultures (CTR), tumor cells in tumor mass developed upon xenografting *in vivo* (X) and xenografted cells recultured *in vitro* for 56 days (De-X)($n_B = 2$; $n_T = 3$). H. Phenotyping of tumor cells (PDOX P3) revealed no changes of marker expression upon bevacizumab treatment *in vivo*.

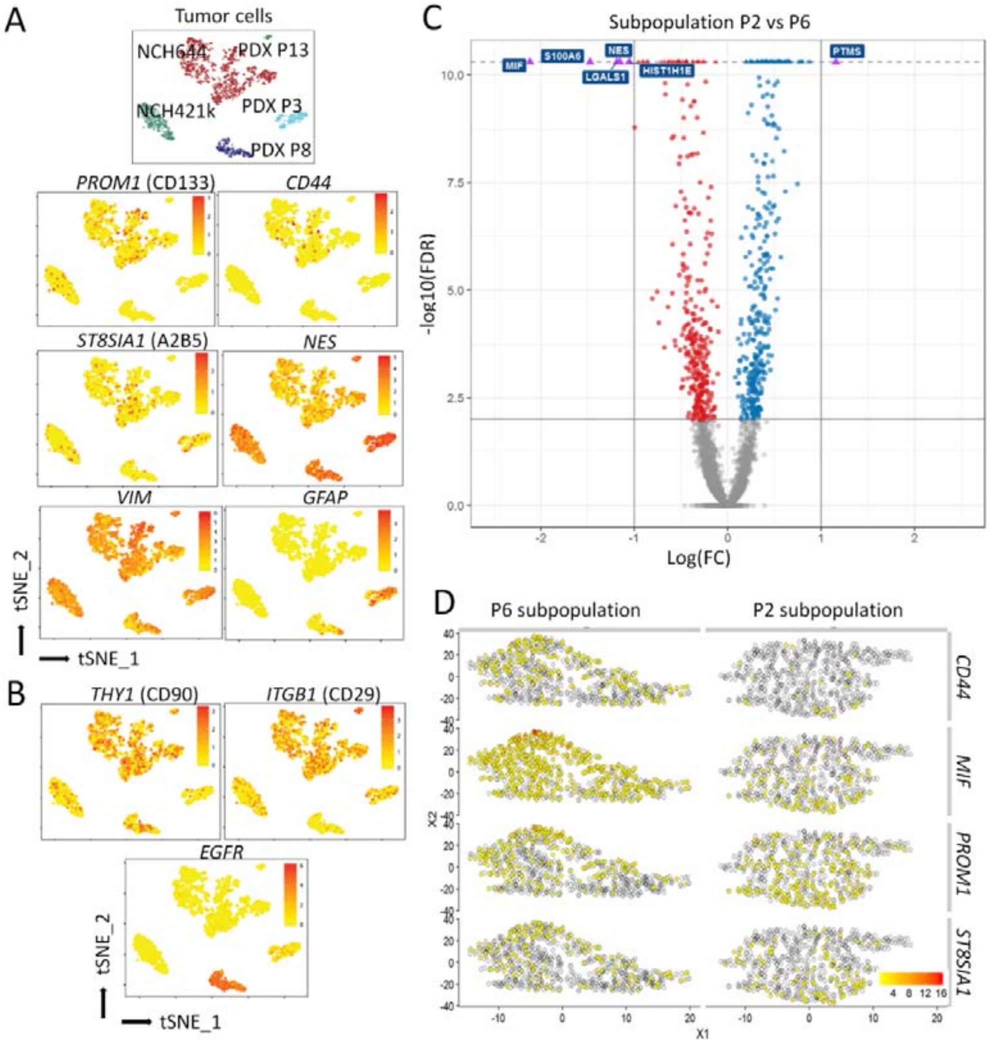

Supplementary Figure 7. Single cell Drop-seq analysis. A-B. tSNE plots showing the expression of marker genes in FACS-sorted tumor cells of 3 GBM PDOXs (P3, P8, P13) and 2 patient derived GBM cultures (NCH644, NCH421k). The expression gradient is color coded. See top-left graph for tSNE plot showing the overall gene expression relationship between single cells of different patient-derived cells. Plots in (A) show the expression of stem cell-associate markers highlighting inter-patient and intratumoral heterogeneity. Plots in (B) show the expression of cell membrane markers (CD90, CD29) with high levels in most tumors and relatively uniform expression profile as detected by flow cytometry. *EGFR* is presented as a control expected to be highly expressed by P8 cells. C. Differential gene expression analysis between single cells of subpopulation P2 versus P6. Threshold for differential expression was set at FDR < 0.01 and |logFC| > 0.5 (DESeq2). Only 6 genes were found to be differentially expressed between the 2 subpopulations (FDR < 0.01, |logFC| > 1). D. tSNE plots showing the expression of marker genes in FACS-sorted subpopulations P2 and P6. The expression gradient is color coded. For visualization purposes cells with no reads were displayed as transparent. *MIF* and *CD44* were expressed at higher levels in FACS-sorted P6 subpopulation. *PROM1* and *ST8SIA1* were detected in a similar proportion of cells. P6 showed upregulation of *Macrophage migration inhibitory factor (MIF),* a known ligand of the CD74/CD44-receptor complex [2]. *CD44* mRNA, the phenotypic discriminator between two subpopulations, was also detected at higher, though not significant, level in the CD44+ P6 subpopulation.

			Equilibrium calculated from single marker state transitions	Equilibrium calculated from four markers state transitions
A. Marker dependency in normoxia				
	CD133+	CD133-		
CD133+	97.48	2.52	77.18	79
CD133-	8.52	91.48	22.82	21
	CD44+	CD44-		
CD44+	95.96	4.04	46.17	41
CD44-	3.46	96.54	53.83	59
	A2B5+	A2B5-		
A2B5+	79.69	20.31	42.91	41.6
A2B5-	15.27	84.73	57.09	58.4
	CD15+	CD15-		
CD15+	93.11	6.89	36.43	39.5
CD15-	3.95	96.05	6.35	60.5
B. Marker dependency in hypoxia				
	CD133+	CD133-		
CD133+	99.7	0.3	97.63	96.19
CD133-	12.4	87.6	2.37	3.81
	CD44+	CD44-		
CD44+	98.87	1.13	73.2	55.79
CD44-	3.09	96.91	26.79	44.21
	A2B5+	A2B5-		
A2B5+	94.05	5.95	40.68	33.92
A2B5-	4.08	95.92	59.32	66.08
	CD15+	CD15-		
CD15+	92.45	7.55	16.27	18.29
CD15-	1.47	98.53	83.73	81.71

Supplementary Table 1. Calculation of marker dependency. Table presents transition probabilities considering only a single marker. A. Marker dependency in normoxia. Single marker equilibria were calculated either by the assumption of independency by adding probabilities of single marker transition matrixes or deviated from the predicted proportions by Markov modelling of 4 markers simultaneously. As equilibria did not differ, markers appeared to be independent. Related to Figure 2E B. Marker dependency in hypoxia. As equilibria did not differ for CD133, A2B5 and CD15, markers appear to be independent. However, CD44 might depend on the combination of CD133, A2B5 and CD15 expression profiles. Related to Figure 3E.

Marker	Gene Symbol				
Cell line		NCH644			
		Hypoxia 12h	Hypoxia 7d	In Vivo	TMZ
CD133	*PROM1*	2.3456	2.3259	-13.231	
CD15	*FUT4*				
CD44	*CD44*	1.52383	1.69996	-10.41035	
A2B5	*ST8SIA1*	-1.54365	-1.67481	-6.4233385	
Nestin	*NES*	1.49565		-2.7380396	
Vimentin	*VIM*	1.89968	2.04294	-2.4077685	
ß-III-Tubulin	*TUBB3*	1.40647			
GFAP	*GFAP*			4.66337	
Cell line		NCH421k			
		Hypoxia 12h	Hypoxia 7d	Xenograft	TMZ
CD133	*PROM1*	2.3353	1.7662	-2.1402	
CD15	*FUT4*				
CD44	*CD44*				
A2B5	*ST8SIA1*	-2.18083	-2.61038		-1.1892
Nestin	*NES*	-1.33667	-2.30914		
Vimentin	*VIM*	1.81312	1.96727	-3.25216	-1.33
ß -III-Tubulin	*TUBB3*			-1.47795	
GFAP	*GFAP*			4.61957	

Supplementary Table 2. Gene expression changes of CSC-associated genes upon exposure to hypoxia, in vivo or TMZ treatment. Expression of CSC-associated genes was analysed within DEG lists for short term hypoxia (12h), long term hypoxia (7d), in vivo and TMZ treatment (TMZ) versus normoxia. Differentially expressed genes between conditions were determined with ANOVA or the eBayes/LIMMA. Cut-off was set up for FDR<0.01 (any fold change). Fold changes are presented only for the significantly altered genes (FDR< 0.01).

Epitope	Conjugate	Clone	Supplier	Concentration
A2B5	APC/PE	105-HB29	Miltenyi	FC:10µl/test
A2B5	AF488	MAB312RX	Chemicon	ICC:1:20
CD15/SSEA-1	AF647	MC-480	Biolegend	FC:5µl/test<br>ICC:1:50
CD15/SSEA-1	PE	MEM-158	Immunotools	FC:10µl/test
CD15/SSEA-1	PerCP-Cy5.5	W6D3	Biolegend	FC:5µl/test
CD24	PE	SN3	Immunotools	FC:10µl/test
CD29	APC	MEM-101A	Immunotools	FC:10µl/test
CD44	FITC	MEM-85	Immunotools	FC: 10µl/test
CD44	PE-Cy7	IM7	eBioscience	FC:1.2µl/test<br>ICC:1:50
CD56	PE-Cy7	N-CAM	BD Bioscience	FC:5µl/test
CD90	PE-Cy7/APC	5E 10	BD Bioscience	FC:5µl/test
CD95	APC	Fas/APO1	BD Bioscience	FC:20µl/test
CD133-1	PE /APC	293C3/AC133	Miltenyi	FC:10µl/test<br>ICC:1:50
CD195	PE	2D7/CCR5	BD Bioscience	FC:20µl/test
EGFR	PE	EGFR.1	BD Bioscience	FC:20µl/test
GFAP	AF647	1B4	BD Bioscience	FC:5µl/test
Isotype control IgG1	FITC	PPV-06	Immunotools	FC:5µl/test
Isotype control IgG1κ	PerCP-Cy5.5	MOPC-21	BD Bioscience	FC:5µl/test
Isotype control IgG2a	AF647	eBR2a	eBioscience	FC:5µl/test
Isotype control IgG2b	AF647	eB1491/10H5	eBioscience	FC:5µl/test
NG2	PE	LHM-2	R&D	FC:10µl/test
Nestin	PerCP-Cy5.5	25/NESTIN	BD Bioscience	FC:5µl/test
Nestin		10C2	Millipore	ICC: 1:200
Vimentin	FITC	V9	Thermo Fischer	FC:5µl/test
Vimentin		V10	Millipore	ICC: 1:200
B-III-tubulin	AF647	TUJ1	BD Bioscience	FC:5µl/test
B-III-tubulin		Tu-20	Millipore	ICC: 1:200
Goat anti-mouse IgG Alexa488	AF488	polyclonal	Thermo Fischer	ICC: 1:500
Goat anti-rabbit IgG Alexa488	AF488	polyclonal	Thermo Fischer	ICC: 1:500
Annexin V	APC		Immunotools	FC: 10µl/test

Supplementary Table 3. Antibodies used in the study. FC = Flow cytometry (test 106 cells/100µl); ICC = Immunocytochemistry

SUPPLEMENTARY REFERENCES

1. Bougnaud, S. *et al.* Molecular crosstalk between tumour and brain parenchyma instructs histopathological features in glioblastoma. *Oncotarget* (2016).
2. Shi, X. *et al.* CD44 is the signaling component of the macrophage migration inhibitory factor-CD74 receptor complex. *Immunity* 25, 595-606 (2006).
