# Supplementary material for: Stem cell-associated heterogeneity in Glioblastoma results from intrinsic tumor plasticity shaped by the microenvironment
Source: Nat Commun. 2019 Apr 16;10:1787. doi: 10.1038/s41467-019-09853-z (PMC6467886; doi:10.1038/s41467-019-09853-z)
Supplement: Supplementary file 3 — Description of Additional Supplementary Files [file 41467_2019_9853_MOESM3_ESM.pdf]

Description of Additional Supplementary Information

File Name: Supplementary Data 1
Description: Statistical analysis of phenotypic states distribution upon treatment and adaptation to changing environment.

File Name: Supplementary Data 2
Description: Statistican analysis of phenotypic state transitions of FACS-sorted subpopulation upon time.

File Name: Supplementary Data 3
Description: Statistican analysis of phenotypic state transitions of FACS-sorted subpopulation upon self-renewal consecutive passages.

File Name: Supplementary Data 4
Description: Statistical analysis of self-renewal potential of 16 FACS-sorted subpopulation in hypoxia.
