# Supplementary material for: Stem cell-associated heterogeneity in Glioblastoma results from intrinsic tumor plasticity shaped by the microenvironment
Source: Nat Commun. 2019 Apr 16;10:1787. doi: 10.1038/s41467-019-09853-z (PMC6467886; doi:10.1038/s41467-019-09853-z)
Supplement: Supplementary file 8 — Reporting Summary [file 41467_2019_9853_MOESM8_ESM.pdf]

nature research

Corresponding author(s): Simone P. Niclou

Last updated by author(s): Mar 13, 2019

Reporting Summary

Nature Research wishes to improve the reproducibility of the work that we publish. This form provides structure for consistency and transparency in reporting. For further information on Nature Research policies, see Authors & Referees and the Editorial Policy Checklist.

Statistics

For all statistical analyses, confirm that the following items are present in the figure legend, table legend, main text, or Methods section.

n/a	Confirmed	
☐	☒	The exact sample size (*n*) for each experimental group/condition, given as a discrete number and unit of measurement
☐	☒	A statement on whether measurements were taken from distinct samples or whether the same sample was measured repeatedly
☐	☒	The statistical test(s) used AND whether they are one- or two-sided<br>*Only common tests should be described solely by name; describe more complex techniques in the Methods section.*
☐	☒	A description of all covariates tested
☐	☒	A description of any assumptions or corrections, such as tests of normality and adjustment for multiple comparisons
☐	☒	A full description of the statistical parameters including central tendency (e.g. means) or other basic estimates (e.g. regression coefficient) AND variation (e.g. standard deviation) or associated estimates of uncertainty (e.g. confidence intervals)
☐	☒	For null hypothesis testing, the test statistic (e.g. *F*, *t*, *r*) with confidence intervals, effect sizes, degrees of freedom and *P* value noted<br>*Give P values as exact values whenever suitable.*
☐	☒	For Bayesian analysis, information on the choice of priors and Markov chain Monte Carlo settings
☐	☒	For hierarchical and complex designs, identification of the appropriate level for tests and full reporting of outcomes
☐	☒	Estimates of effect sizes (e.g. Cohen's *d*, Pearson's *r*), indicating how they were calculated

Our web collection on statistics for biologists contains articles on many of the points above.

Software and code

Policy information about availability of computer code

Data collection	No software was used in the study
Data analysis	Scripts supporting mathematical modeling are available in the freely available R package CellTrans (http://github.com/tbuder/CellTrans)

For manuscripts utilizing custom algorithms or software that are central to the research but not yet described in published literature, software must be made available to editors/reviewers. We strongly encourage code deposition in a community repository (e.g. GitHub). See the Nature Research guidelines for submitting code & software for further information.

Data

Policy information about availability of data

All manuscripts must include a data availability statement. This statement should provide the following information, where applicable:

- Accession codes, unique identifiers, or web links for publicly available datasets
- A list of figures that have associated raw data
- A description of any restrictions on data availability

The gene expression patterns across glioma patients were investigated using publicly available datasets: (i) GlioVis data portal http://gliovis.bioinfo.cnio.es/; (ii) Cbioportal http://www.cbioportal.org/; and (iii) RecurR http://recur.bioinfo.cnio.es/. Microarray data are available in the ArrayExpress database (https://www.ebi.ac.uk/arrayexpress/) under the accession number E-MTAB-3085. The scRNA-seq data are available in the Gene Expression Omnibus (https://www.ncbi.nlm.nih.gov/geo) under the accession number GSE128195. Scripts supporting mathematical modeling are available in the R package CellTrans (http://github.com/tbuder/CellTrans). Remaining datasets supporting the findings are available from the corresponding author on reasonable request.

Field-specific reporting

Please select the one below that is the best fit for your research. If you are not sure, read the appropriate sections before making your selection.

☒ Life sciences ☐ Behavioural & social sciences ☐ Ecological, evolutionary & environmental sciences

For a reference copy of the document with all sections, see nature.com/documents/nr-reporting-summary-flat.pdf

Life sciences study design

All studies must disclose on these points even when the disclosure is negative.

Sample size	The number of biological and technical replicates for in vitro studies and mathematical modeling has been based on previous experience with flow cytometry-based experiments and assessed by an experiences statistician. For in vivo tumorigenicity assay a sample size power statistical analysis, using a two group t-test with a 0.05 two-sided significance level, revealed a prerequisite for 7 mice per group.
Data exclusions	No data were excluded from the analyses. All cells/animals that met proper experimental conditions were included in the analysis.
Replication	All experimental findings were reliable reproduced.
Randomization	No randomisation was used to allocate experimental groups.
Blinding	To reduce the bias the in vivo tumorigenicity test was double-blinded for the animal take carers.

Reporting for specific materials, systems and methods

We require information from authors about some types of materials, experimental systems and methods used in many studies. Here, indicate whether each material, system or method listed is relevant to your study. If you are not sure if a list item applies to your research, read the appropriate section before selecting a response.

Materials & experimental systems

n/a	Involved in the study
☐	☒ Antibodies
☐	☒ Eukaryotic cell lines
☒	☐ Palaeontology
☐	☒ Animals and other organisms
☒	☐ Human research participants
☒	☐ Clinical data

Methods

n/a	Involved in the study
☒	☐ ChIP-seq
☐	☒ Flow cytometry
☒	☐ MRI-based neuroimaging

Antibodies

Antibodies used

The following antibodies were used in the study (Epitope, Conjugate, Clone, Supplier, Concentration)
A2B5 APC/PE 105-HB29 Miltenyi FC:10µl/test
A2B5 AF488 MAB312RX Chemicon ICC:1:20
CD15/SSEA-1 AF647 MC-480 Biolegend FC:5µl/test ICC:1:50
CD15/SSEA-1 PE MEM-158 Immunotools FC:10µl/test
CD15/SSEA-1 PerCP-Cy5.5 W6D3 Biolegend FC:5µl/test
CD24 PE SN3 Immunotools FC:10µl/test
CD29 APC MEM-101A Immunotools FC:10µl/test
CD44 FITC MEM-85 Immunotools FC: 10µl/test
CD44 PE-Cy7 IM7 eBioscience FC:1.2µl/test ICC:1:50
CD56 PE-Cy7 N-CAM BD Bioscience FC:5µl/test
CD90 PE-Cy7/APC 5E 10 BD Bioscience FC:5µl/test
CD95 APC Fas/APO1 BD Bioscience FC:20µl/test
CD133-1 PE /APC 293C3/AC133 Miltenyi FC:10µl/test ICC:1:50
CD195 PE 2D7/CCR5 BD Bioscience FC:20µl/test
EGFR PE EGFR.1 BD Bioscience FC:20µl/test
GFAP AF647 1B4 BD Bioscience FC:5µl/test
Isotype control IgG1 FITC PPV-06 Immunotools FC:5µl/test
Isotype control IgG1κ PerCP-Cy5.5 MOPC-21 BD Bioscience FC:5µl/test
Isotype control IgG2a AF647 eBR2a eBioscience FC:5µl/test
Isotype control IgG2b AF647 eB1491/10H5 eBioscience FC:5µl/test
NG2 PE LHM-2 R&D FC:10µl/test
Nestin PerCP-Cy5.5 25/NESTIN BD Bioscience FC:5µl/test
Nestin 10C2 Millipore ICC: 1:200

Vimentin FITC V9 Thermo Fischer FC:5µl/test
Vimentin V10 Millipore ICC: 1:200
B-III-tubulin AF647 TUJ1 BD Bioscience FC:5µl/test
B-III-tubulin Tu-20 Millipore ICC: 1:200
Goat anti-mouse IgG Alexa488 AF488 polyclonal Thermo Fischer ICC: 1:500
Goat anti-rabbit IgG Alexa488 AF488 polyclonal Thermo Fischer ICC: 1:500
AnnexinV Immunotools FC: 10µl/test

Validation	Each antibody was validated and titrated if required by flow cytometry and/or immunocytochemistry based on standard validation protocols.

Eukaryotic cell lines

Policy information about cell lines

Cell line source(s)	The GBM serum-free cultures NCH421k, NCH660h, NCH465, NCH601 and NCH644, were provided by Dr Christel Herold-Mende (Department of Neurosurgery, University of Heidelberg). The GBM cultures TB101 and TB107, kindly provided by Dr. Håkan Hedman, (Umeå University, Sweden). U87 cells were obtained from ATCC (HTB-14).
Authentication	Cell lines used within 2 years since obtaining them from the source laboratory were not authenticated (TB101, TB107). Other cell lines were authenticated by DSMZ using SNP-based multiplex approach. SNP profiles were unique.
Mycoplasma contamination	All cell lines were regularly tested for mycoplasma contamination.
Commonly misidentified lines (See ICLAC register)	None of the cell lines used are listed in the ICLAC list.

Animals and other organisms

Policy information about studies involving animals; ARRIVE guidelines recommended for reporting animal research

Laboratory animals	eGFP-expressing NOD/Scid mice and nude mice were used in the study. The sex and age was standardized for the animals used for the in vivo tumorigenicity study.
Wild animals	The study did not involve wild animals.
Field-collected samples	The study did not involve samples collected from the field.
Ethics oversight	The handling of the animals and the surgical procedures were performed in accordance with the European Directive on animal experimentation (2010/63/EU) and the local ethical committees approved the protocol (Animal Welfare Structure of the LIH).

Note that full information on the approval of the study protocol must also be provided in the manuscript.

Flow Cytometry

Plots

Confirm that:

☒ The axis labels state the marker and fluorochrome used (e.g. CD4-FITC).

☒ The axis scales are clearly visible. Include numbers along axes only for bottom left plot of group (a 'group' is an analysis of identical markers).

☒ All plots are contour plots with outliers or pseudocolor plots.

☒ A numerical value for number of cells or percentage (with statistics) is provided.

Methodology

Sample preparation	Please see the Material and Methods section for detailed protocols.
Instrument	Data acquisition was performed on a FACS AriaTM SORP cytometer (BD Biosciences) and ImageStream imaging cytometer (Amnis).
Software	Data acquisition and analysis were done with DIVA software (BD Bioscience). Histograms were prepared with the FlowJo software. ImageStream-based acquisition was performed with the INSPIRE® software and analysis was performed using IDEAS® image analysis software.
Cell population abundance	The purity of the sorted samples war regularly tested. Only populations with >97% of purity were further applied for experiments.
Gating strategy	Gating strategy included exclusion of debris, doublets, dead cells and anucleated events (intracellular staining if applicable).

Gating strategy | Gating strategy is presented in Figure S2

☒ Tick this box to confirm that a figure exemplifying the gating strategy is provided in the Supplementary Information.
